# Radioimmunotherapy combating biofilm-associated infection *in vitro*

**DOI:** 10.3389/fmed.2024.1478636

**Published:** 2024-11-29

**Authors:** Zijian Ye, Berend van der Wildt, F. Ruben H. A. Nurmohamed, J. Fred F. Hooning van Duyvenbode, Jos van Strijp, H. Charles Vogely, Marnix G. E. H. Lam, Ekaterina Dadachova, Harrie Weinans, Bart C. H. van der Wal, Alex J. Poot

**Affiliations:** ^1^Department of Orthopaedics, University Medical Center Utrecht, Utrecht, Netherlands; ^2^Department of Radiology and Nuclear Medicine, University Medical Center Utrecht, Utrecht, Netherlands; ^3^Department of Medical Microbiology, University Medical Center Utrecht, Utrecht, Netherlands; ^4^College of Pharmacy and Nutrition, University of Saskatchewan, Saskatoon, SK, Canada; ^5^Department of Biomechanical Engineering, Faculty of Mechanical Engineering, Delft University of Technology, Delft, Netherlands

**Keywords:** radioimmunotherapy, *Staphylococcus aureus*, biofilm, antibodies, infection, wall teichoic acids, actinium-225, lutetium-177

## Abstract

**Background:**

Addressing prosthetic joint infections poses a significant challenge within orthopedic surgery, marked by elevated morbidity and mortality rates. The presence of biofilms and infections attributed to *Staphylococcus aureus* (*S. aureus*) further complicates the scenario.

**Objective:**

To investigate the potential of radioimmunotherapy as an innovative intervention to tackle biofilm-associated infections.

**Methods:**

Our methodology involved employing specific monoclonal antibodies 4497-IgG1, designed for targeting wall teichoic acids found on *S. aureus* and its biofilm. These antibodies were linked with radionuclides actinium-225 (^225^Ac) and lutetium-177 (^177^Lu) using DOTA as a chelator. Following this, we evaluated the susceptibility of *S. aureus* and its biofilm to radioimmunotherapy *in vitro*, assessing bacterial viability and metabolic activity via colony-forming unit enumeration and xylenol tetrazolium assays.

**Results:**

Both [^225^Ac]4497-IgG1 and [^177^Lu]4497-IgG1 exhibited a noteworthy dose-dependent reduction in *S. aureus* in planktonic cultures and biofilms over a 96-h exposure period, compared to non-specific antibody control groups. Specifically, doses of 7.4 kBq and 7.4 MBq of [^225^Ac]4497-IgG1 and [^177^Lu]4497-IgG1 resulted in a four-log reduction in planktonic bacterial counts. Within biofilms, 14.8 kBq of [^225^Ac]4497-IgG1 and 14.8 Mbq [^177^Lu]4497-IgG1 led to reductions of two and four logs, respectively.

**Conclusion:**

Our findings underscore the effectiveness of [^225^Ac]4497-IgG1 and [^177^Lu]4497-IgG1 antibodies in exerting dose-dependent bactericidal effects against planktonic S. *aureus* and biofilms *in vitro*. This suggests that radioimmunotherapy might serve as a promising targeted treatment approach for combating S. *aureus* and its biofilm.

## 1 Introduction

The management of prosthetic joint infection (PJI) is an important challenge in orthopedic surgery and significantly impacts patient morbidity and mortality (1, 2). With the increasing number of joint replacement surgeries worldwide, the incidence of PJI is increasing (3). This is of particular concern for elderly patients, as they are more susceptible to complications, which would result in a greater economic burden on healthcare systems (4). These infections pose challenges in treatment due to the development of biofilms on artificial surfaces and the presence of bacteria resistant to antibiotics, such as methicillin-resistant *Staphylococcus aureus* (MRSA) (5). Biofilms are structured communities of microorganisms that produce a naturally occurring extracellular polymeric substance (EPS) network. This network shields enclosed cells from antimicrobial substances and the host immune response, thereby extending the duration of infection and complicating efforts to eliminate it (6). Consequently, there is an increasing need for alternative approaches to therapy.

In the past, ionizing radiation was employed in the treatment of bacterial infections prior to the introduction of antibiotics. Although external radiation demonstrated certain efficacy before the antibiotic era, its extensive application was hindered by significant side effects and challenges in precisely targeting pathogens without damaging surrounding tissues (7). These discoveries underscore the necessity for more precise approaches in contemporary medical practice of bactericidal radiation therapy.

An innovative option on the horizon is radioimmunotherapy (RIT), which merges the precision of monoclonal antibodies (mAbs) with the cytotoxic power of radionuclides. RIT represents a novel therapeutic strategy, successfully deployed against various cancers by leveraging mAbs ability to selectively pinpoint tumor cells while sparing healthy tissue (8). Extending this concept to infections is a recent development. Dadachova et al., demonstrated RIT’s efficacy in combatting fungal and bacterial infections (9, 10). This targeting approach hinges on recognizing antigens expressed uniquely or significantly overexpressed on the surface of organisms.

*In vitro* and *in vivo* experiments demonstrated the efficacy of 4497-IgG1 (anti-β-GlcNAc WTA) as a highly efficient vector for targeted transportation, as shown earlier by de Vor and van Dijk (11, 12). Wall teichoic acids (WTA) are anionic polymers covalently bound to the peptidoglycan layer found in the cell walls of many Gram-positive bacteria, including S. *aureus* and MRSA, and in the extracellular matrix of biofilms (13). Monoclonal antibodies targeting WTA can specifically direct the cytotoxic effects of radionuclides to *S. aureus*, including those within biofilms. Additionally, the potential of the alpha emitter bismuth-213 was demonstrated with mAbs against WTA using MRSA biofilm culture *in vitro*, but its short half-life (45.6 mins) limits its application (14). Therefore, we selected actinium-225 (^225^Ac, an α-emitter, with t_1/2_ = 9.92 days) and lutetium-177 (^177^Lu, a β-emitter, with t_1/2_ = 6.7 days) as particularly attractive choices due to their unique decay properties. Alpha emitters release high-energy particles that can effectively kill bacteria in a localized manner, minimizing damage to surrounding healthy tissue. Beta emitters provide a wider range of radiation, allowing for deeper tissue penetration and a broader distribution of therapeutic effects. Combining these radionuclides with mAbs targeting WTA can deliver a local bactericidal effect directly to bacteria and their biofilms. In this study, we investigated the antibacterial effect of RIT on *S. aureus* and its biofilms using [^225^Ac]4497-IgG1 and [^177^Lu]4497-IgG1.

## 2 Materials and methods

### 2.1 Bacterial strain and growth conditions

*Staphylococcus aureus* USA300 LAC (AH4802) strain was used (12). Bacteria were cultured overnight at 37°C on sheep blood agar (SBA) medium and subsequently transferred to tryptone soy broth (TSB) for further overnight culture. Prior to each experiment, overnight cultured bacteria were re-inoculated into fresh TSB medium and incubated for 3 h to allow for a logarithmic growth period to ensure that the bacteria were in an optimal growth state for subsequent experimental manipulation and data accuracy.

### 2.2 Biofilm culture and crystal violet staining

The overnight cultured bacteria were adjusted to an OD600 of 1.0 with fresh TSB solution containing 0.5% glucose and 3% NaCl and then diluted 1000-fold. Next, 200 μl of the bacterial suspension was taken and added to the wells of Corning^®^ 96 Well TC-Treated Microplates and left to incubate for 24 h at 37°C. To enhance the adhesion of bacteria to the well plates, the well plates were treated with 20 μg/mL of bovine fibronectin (Sigma) in 0.1 M carbonate-bicarbonate buffer (pH 9.4 - 9.8) at 4°C overnight prior to the experiment. The biofilm formation was validated via crystal violet staining. Wells were carefully washed three times with PBS to remove non-adherent cells, and adherent biofilms were fixed by drying the plate at 60°C for 1 h. Adherent cells were stained with 0.1% crystal violet for 5 min, and excess dye was removed by rinsing with distilled water. To quantify biofilm biomass, the remaining crystal violet was solubilized in 33% acetic acid, and the absorbance was measured at 595 nm using a CLARIOstar plate reader (BMG LABTECH).

### 2.3 Radionuclides, chelators and antibodies

Lutetium chloride NCA (^177^LuCl_3_) was obtained from ITM (Munich, Germany), and actinium nitrate (^225^AcNO_3_) was obtained from VON (Breda, The Netherlands). The Anti-β-GlcNAc WTA antibody 4497-IgG1 was synthesized following the protocol described elsewhere (12). Briefly, the variable heavy (VH) and light (VL) chain sequences were cloned into pcDNA3.4 vectors containing human constant regions. These sequences, originally from B cells of *S. aureus*-infected patients, were codon optimized and included KOZAK sequences and HAVT20 signal peptides. Transfected into EXPI293F cells, IgG1 antibodies were purified from the supernatant using HiTrap protein A columns after 4–5 days. The antibodies were then dialyzed in PBS, sterilized via filtration, checked for aggregation, and stored at 4.8 mg/mL concentration at −20°C. Dinutuximab (Qarziba) was used as a control antibody. The bifunctional chelator, benzyl 4-isothiocyanatobenzyl 1,4,7,10-tetraazacyclododecane-N′, N′′, N′′, N′′ tetraacetic acid (p -SCN-Bz-DOTA) was purchased from Macrocyclics Inc., in Richardson, Texas (Stock No. B205).

### 2.4 Susceptibility of *S.aureus* and biofilm to free radionuclides

To assess the susceptibility of *S. aureus* and its biofilm to non-targeted radionuclides, bacterial cultures were grown in TSB. The study used different doses of radionuclides, 0 to 14.8 kBq ^225^Ac and 0 to 7.4 MBq of ^177^Lu, to treat biofilm and planktonic forms of *S. aureus*. These dose levels were chosen based on existing literature and preliminary experiments, demonstrating their effectiveness for significant impacts on bacterial growth and viability without causing immediate, overwhelming lethality (14). Biofilm cultures were established with an initial density of 10^7^ CFU/ml in 96 well plates, while planktonic cultures were started at 10^8^ CFU/ml in 1.5ml microcentrifuge tubes. Cultures were exposed to radionuclides in a thermomixer at 600 rpm at 37 degrees Celsius for up to 168 h and were examined for radionuclide sensitivity at each specified time interval. The 168-h exposure period was selected to correspond with the half-lives of ^225^Ac (9.92 days) and ^177^Lu (6.7 days), allowing for comprehensive observation of immediate and prolonged effects. The effect of radionuclides on bacterial growth and viability was monitored as determined by CFU counting. The results are expressed as mean CFU count ± standard deviation (*n* = 3).

### 2.5 Conjugation and radiolabeling

At least 2 mg of 4497-IgG1 antibody at a concentration of 4.8 mg/ml was added to a 30K MWCO Amicon Ultra 0.5 mL centrifugal filter (Millipore). 10 washes of 0.3 ml each were performed using a cooled centrifuge at 4°C with 0.1 M NaHCO3 buffer (pH 9) for buffer change. After buffer change, the antibody was coupled with an excess of p-SCN-Bz-DOTA at a molar ratio of 1:10 and incubated for 30 min at 37°C in a thermostatic mixer. After incubation, the coupling was purified with another Amicon filter and 0.25 M ammonium acetate buffer (pH 5–5.5) to remove unreacted p-SCN-Bz-DOTA. Finally, the coupling was re-suspended in 300 μl of ammonium acetate buffer and stored in metal-free tubes at 4°C until radiolabeling.

Radiolabeling was performed in a metal-free environment. Approximately 1 MBq of actinium-225 chloride or 500 MBq of lutetium-177 chloride was transferred to a glass vial and diluted to 1 ml with 0.5 M ammonium acetate buffer (pH 5–5.5). The antibody-DOTA conjugate was adjusted to a volume of 1 mL using the same buffer, ensuring a concentration of antibodies of at least 1 mg/ml. Both components were incubated at 42°C for 1 h in a heating block. The mixture was then processed through a PD-10 size-exclusion column, pre-washed with 5 mg/ml ascorbic acid in PBS. The conjugate was eluted with 0.5 ml fractions of the same ascorbic acid solution, collecting fractions based on their radioactivity. Fractions were pooled as necessary, and the purity of the radiolabeled conjugate was then assessed.

### 2.6 Radiochemical purity test

The radiochemical purity of the conjugate was analyzed using instant thin-layer chromatography (iTLC). Briefly, a 1 μl sample of the product was applied to a silica-coated TLC paper strip (Agilent Technologies, SGI0001) at 2 cm from the bottom. The strip was eluted using a mixture of 0.01 M EDTA and acetonitrile (50:50, v/v). After development, the strips were cut into ten equal parts. The radioactivity of each fragment was measured using a gamma counter. The counts recorded for each fragment were summed and averaged to assess the radiochemical purity of the labeled compounds.

### 2.7 Immunoreactivity assay

To assess the immunoreactivity of radiolabeled mAbs, 1.5 ml microcentrifuge tubes were first blocked with 1% bovine serum albumin (BSA) in PBS for 1 h to inhibit non-specific binding. A series of eight serial dilutions of a bacterial suspension was prepared in 0.1% BSA in PBS, with final antibody concentrations adjusted to 4 μg/mL. Following incubation at 37°C for 60 min on a thermomixer set to 600 rpm, samples were centrifuged at 17,000g for 4 min, and the supernatants were transferred to new tubes. The samples were washed twice with PBS. A gamma counter was used to measure radioactivity in pellets and supernatant, indicating the percentage of ^225^Ac or ^177^Lu bound to the cells and the percentage of free ^225^Ac or ^177^Lu, respectively. The cell binding fraction was determined as the radioactivity in pellets ratio to total radioactivity. A double inverse plot of total applied activity divided by the inverse ratio of cell binding activity to cell concentration was drawn, and a linear curve was fitted to the data points. According to the method described by Lindmo et al., the reciprocal of the curve intercept was determined and treated as immune reactivity (representing the case of infinite antigen excess) (15).

### 2.8 Determination of RIT antibacterial effect against *S. aureus* and biofilm

To assess the *in vitro* antimicrobial efficacy of RIT against *S. aureus* and biofilms, mAb 4497-IgG1 labeled with ^225^Ac or ^177^Lu was used. Dinutuximab was radiolabeled in a similar manner and served as an isotype control. Bacteria were incubated in PBS containing 10–40 μg of [^225^Ac]mAb (1.85–14.8 kBq) or [^177^Lu]mAb (1.85–14.8 MBq) for 60 min at 37°C to allow the radiolabeled antibody to bind to the cell surface. As a control, bacteria were also incubated in PBS containing 20 μg DOTA-coupled unlabeled mAbs and free radionuclides of the same radioactivity. After 60-min binding incubation, unbound radioactivity was removed by centrifugation at 17,000 g for 4 mins, and the bacterial precipitate was washed and resuspended in PBS and kept on a shaker at 4°C for 96 h exposure. The bacteria were then incubated in PBS and incubated with the same mAbs and free radionuclides. This nutrient-free condition prevented bacterial division during the incubation and maintenance phases. Samples were taken and counted in a gamma counter to verify that the radioactivity remained attached to the cells after 96 h of incubation. The sample was then centrifuged to separate the cell precipitate and supernatant, washed with PBS and counted again in a gamma counter. Afterward, the tubes were centrifuged at 17,000 g for 4 mins, washed twice, and the microspheres were resuspended in 100 microliters of sterile PBS. Biofilms were disrupted by sonication for 60 s to aid in cell separation. Bacterial cell and biofilm viability and metabolic activity were determined using CFU counting and XTT reduction assay.

### 2.9 Colony forming units counting assay and XXT assay

Serial dilutions (10 μl each) of the bacterial suspension with PBS were plated on blood agar and incubated overnight, with colonies counted to assess viability. Metabolic activity was assessed by adding 50 μl of freshly prepared 2,3-bis(2-methoxy-4-nitro-5-sulfophenyl)-5-[(phenylamino)carbonyl]-2H-tetrazolium hydroxide (XTT) solution to 80 μl of the bacterial suspension and incubated statically at 37°C for 3 h. The colorimetric changes, indicative of mitochondrial dehydrogenase activity, were measured at an absorbance of 492 nm using a CLARIOstar Plus microplate reader. Blank controls consisted of two wells containing sterile PBS.

### 2.10 Statistical analyses

Statistical analyses were conducted using GraphPad Prism 10 software. To compare the effects of different groups and dosages, Analysis of Variance (ANOVA) was employed. Repeated measures ANOVA was utilized to assess changes before and after treatment. The paired *t*-test was used to evaluate the significance of the treatment effects. A *p*-value of less than 0.05 was considered statistically significant. All experiments were performed in triplicate to ensure reproducibility and accuracy.

## 3 Results

### 3.1 Radiochemical purity and immunoreactivity

4497-IgG1 was successfully modified with a DOTA chelator and subsequently radiolabeled with actinium-225 or lutetium-177. [^225^Ac]4497-IgG1 was obtained in 54% yield (540 kBq) and displayed a radiochemical purity of 95.4%. [^177^Lu]4497-IgG1 was obtained in 46% yield (230 MBq) and displayed a radiochemical purity of 96.16%.

**FIGURE 1:**
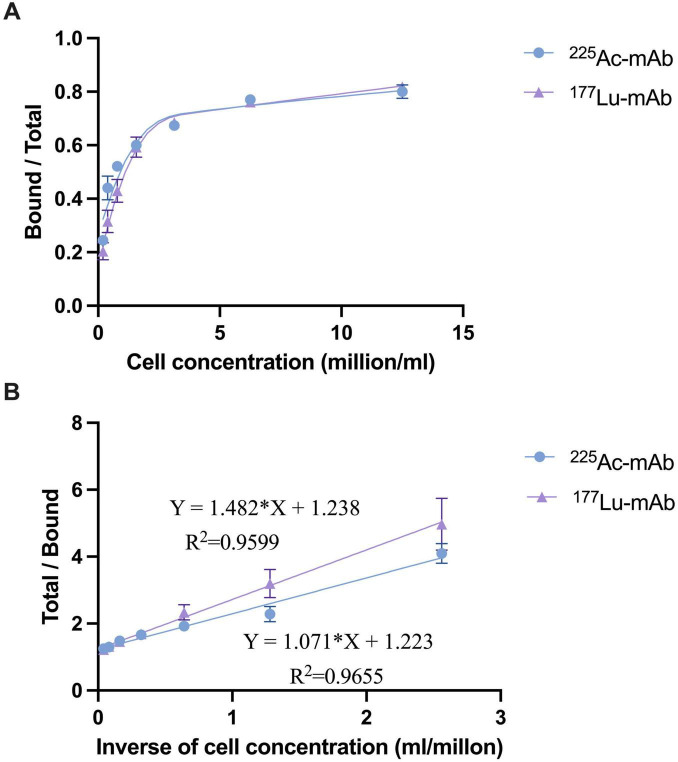
Binding plot **(A)** of the ratio of specifically bound radiolabeled antibody to the total applied radioactivity as a function of cell concentration (*X*-axis). The cell concentration is expressed as cells (million/ml). Lindmo plot **(B)** showing total/bound activity as a function of inverse cell concentration expressed as 1/cells (ml/million).

The immunoreactivity assays demonstrated high binding affinity of the radiolabeled 4497-IgG1 conjugates to target cells, with MRSA retaining approximately 80% of its immunoreactivity at the highest cell concentration (Figure 1A). Analysis of the double reciprocal plot (Scatchard analysis) indicated a linear relationship between total binding and the inverse of cell concentration (Figure 1B). For [^225^Ac]4497-IgG1, this relationship followed the equation *Y* = 1.071*X* + 1.223 with an *R*^2^ value of 0.9655 and an immunoreactivity fraction *r* of 0.818. For [^177^Lu]4497-IgG1, the equation was *Y* = 1.482*X* + 1.238 with an *R*^2^ of 0.9599 and an immunoreactivity fraction *r* of 0.808.

### 3.2 Susceptibility of *S. aureus* and its biofilm to free radionuclides

**FIGURE 2:**
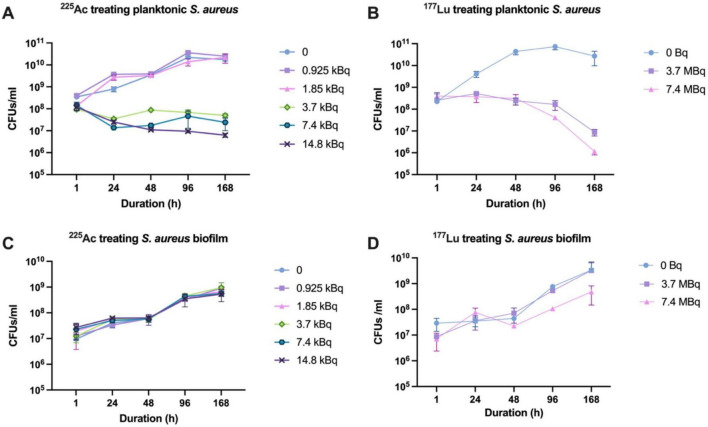
Susceptibility of *S. aureus* and its biofilm to ^225^Ac and ^177^Lu. **(A)** Susceptibility of planktonic *S. aureus* to ^225^Ac. **(B)** Susceptibility of planktonic *S. aureus* to ^177^Lu. **(C)** Susceptibility of *S. aureus* in biofilm state to ^225^Ac. **(D)** Susceptibility of *S. aureus* in biofilm state to ^177^Lu.

Planktonic bacteria exhibited susceptibility to free ^225^Ac, showing growth inhibition when concentrations exceeded 3.7 kBq, although no pronounced lethality was observed (Figure 2A). Similarly, exposure to doses exceeding 3.7 MBq of free ^177^Lu led to bacterial growth inhibition at 96 h, with a reduction in viable counts exceeding 2 logs even at 168 h (Figure 2B). However, bacteria within the biofilm demonstrated marked resistance to both free radionuclides, likely attributed to limited radiation penetration. Not only did the biofilm impede inhibitory effects, but it also increased over time (Figures 2C, D).

### 3.3 Antibacterial efficacy of RIT against *S. aureus* and its biofilm

**FIGURE 3:**
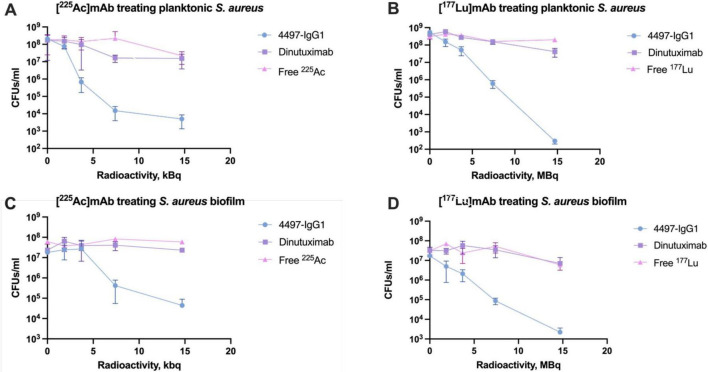
Antibacterial efficacy of RIT against *S. aureus* and its biofilm. **(A)** [^225^Ac]mAb treating planktonic *S. aureus*. **(B)** [^177^Lu]mAb treating planktonic *S. aureus*. **(C)** [^225^Ac]mAb treating *S. aureus* biofilm. **(D)** [^177^Lu]mAb treating *S. aureus* biofilm.

[^225^Ac] 4497-IgG1 and [^177^Lu]4497-IgG1 were assessed for antibacterial efficacy against planktonic *S. aureus* and biofilms. Both radiolabeled 4497-IgG1 mAbs exhibited a dose-dependent relationship with the bacterial load, as illustrated in Figure 3. Control experiments using unlabeled 4497-IgG1 confirmed the absence of bactericidal activity from the antibody itself. In comparison to the non-specific antibody (Dinutuximab) and free radionuclides, radiolabeled specific antibodies significantly reduced bacterial loads within the investigated dose range (*P* < 0.01). Notably, a 7.4 kBq of [^225^Ac] 4497-IgG1 and 7.4 MBq of [^177^Lu]4497-IgG1 resulted in an over 4-log and 6-log reduction in planktonic bacterial numbers (Figures 3A, B).

Interestingly, biofilms demonstrated higher resistance to [^225^Ac]4497-IgG1, with no significant antibacterial effect below 3.7 kBq. At the highest dose of [^225^Ac]4497-IgG1, bacterial counts were reduced by two logarithmic values (Figure 3C). Conversely, [^177^Lu]4497-IgG1 exhibited a robust dose-dependent effect, reducing bacterial counts by up to four logarithmic values (Figure 3D). Nevertheless, both radionuclides demonstrated significantly enhanced antibacterial efficacy compared to control groups (*P* < 0.01).

**FIGURE 4:**
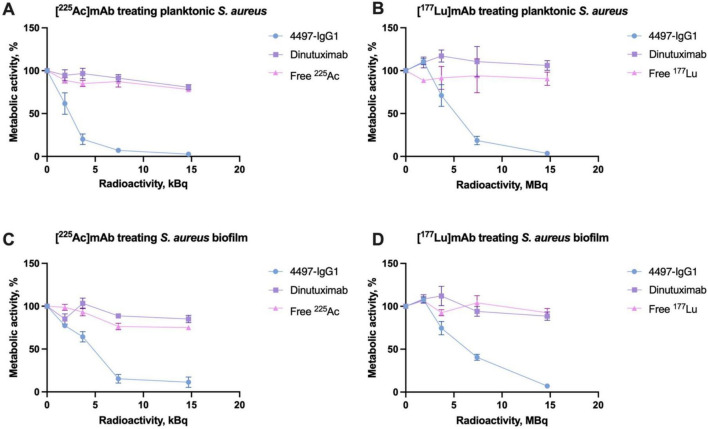
XTT assay results showing the metabolic activity of *S. aureus* and its biofilm after treatment with RIT. **(A)** Metabolic activity of planktonic *S. aureus* treated with [^225^Ac]mAb. **(B)** Metabolic activity of planktonic *S. aureus* treated with [^177^Lu]mAb. **(C)** Metabolic activity of *S. aureus* biofilm treated with [^225^Ac]mAb. **(D)** Metabolic activity of *S. aureus* biofilm treated with [^177^Lu]mAb.

These observations were corroborated by metabolic XTT analysis, revealing a reduction in metabolic activity proportional to bacterial count decreases. Slight increases in metabolic activity at low doses could be attributed to a bacterial stress response and potential DNA repair activation triggered by low-level radiation exposure (Figure 4). Overall, the enhanced bactericidal effect of specific mAbs provides excellent eradication efficacy due to increased localized irradiation following targeted binding.

## 4 Discussion

Biofilm-associated periprosthetic infections pose a significant threat to global health systems, and conventional treatments are inadequate to meet this challenge. We propose the use of radioimmunotherapy against *S. aureus* and its biofilm. The specific binding of 4497-IgG1 to WTA on *S. aureus* and biofilms *in vitro* and *in vivo* has been verified in our previous study (11, 12). Based on this antibody, we developed ^225^Ac and ^177^Lu radiopharmaceuticals optimized to exhibit excellent chemical properties. The radionuclides bind to the antibodies via DOTA chelators, ensuring stable attachment and the ability to target the bacteria effectively. The results indicated that the radiochemical purity of the radio-complexes reached 96%, with an immunoreactivity rate as high as 82%. Moreover, the antibody’s binding capability remained largely unaffected. These observations imply that incorporating radionuclides into the antibody structure did not compromise its functional integrity; rather, it precisely facilitated targeted delivery of radiopharmaceuticals to directly combat bacteria. These outcomes are consistent with previous research, underscoring the efficacy of DOTA chelating agents in maintaining stable radionuclide binding while preserving antibody immunological activity (16, 17).

Here, we reported that [^225^Ac]4497-IgG1 and [^177^Lu]4497-IgG1 demonstrated a dose-response bactericidal impact on free-floating *S. aureus*. Both compounds substantially decreased bacterial survival across the examined dose spectrum relative to the control cohort. The robust bactericidal influence of ^225^Ac at lower radioactivity levels compared to ^177^Lu might stem from α-particles’ higher linear energy transfer (LET) in contrast to β-particles. Each decay of four α-particles emits considerable energy over a brief distance, inducing severe localized damage. This result is consistent with other radioimmunotherapy studies against different bacteria where significant bacterial reduction using radioimmunotherapy have been demonstrated with bismuth-213 and rhenium-188 against *Streptococcus pneumoniae* and *Bacillus anthracis* (10, 18).

When dealing with biofilms, some obstacles exist. Although [^225^Ac]4497-IgG1 and [^177^Lu]4497-IgG1 showed anti-biofilm effects (more than 2-log reduction) (Figure 2), ^177^Lu was more effective. However, the anti-biofilm effect of both radionuclides was lower than their bactericidal effect on planktonic bacteria. This is expected because of the complexity of the biofilm structure, low metabolic activity, and the thick polysaccharide and protein matrix (6). The desired log reduction for effective antimicrobial treatment is typically 99.9% (3-log reduction), which our study did not fully achieve against biofilms. Although alpha particles such as ^225^Ac have a limited range (∼40 μm), sufficient to cover a bacterium and an entire biofilm thickness of approximately 20 μm in this case, a dense arrangement of bacteria within a biofilm impedes penetration of radioactive particles, making it difficult for RIT to penetrate the entire biofilm and leading to eradication of deep-seated bacteria. On the other hand, beta particles such as ^177^Lu have a much longer range (∼2 mm) and stronger penetrability, allowing them to cover the entire biofilm. Therefore, using alpha-emitting radionuclides in combination with biofilm disintegration techniques may help improve overall efficacy. For example, Watters et al., reported that the use of α-amylase, bromelain, and papain could reduce the biomass of MRSA biofilm by more than 97% (19).

The mechanism by which RIT treats bacterial infections involves two main aspects: direct cytotoxicity and the bystander effect. Radiolabeled monoclonal antibodies disrupt bacterial metabolism by delivering radiation directly to the bacteria causing DNA damage (direct effect) and the production of reactive oxygen species (ROS, bystander effect). The damage results in mutations, breaks in the DNA strands, and if the bacteria fail to repair the damage, ultimately leads to cell death (20). The ROS, such as superoxide radicals, hydrogen peroxide, and hydroxyl radicals, contribute to cellular damage by attacking lipids, proteins, and nucleic acids. This impairs important metabolic pathways and energy production. Studies have demonstrated that oxidative stress from ROS significantly inhibits bacterial respiration and metabolic processes, resulting in the accumulation of toxic intermediates and depletion of critical metabolites, ultimately leading to bacterial cell death (21, 22). Furthermore, the bystander effect contributes to the overall efficacy of RIT. Even if bacteria are not directly exposed to radiation, damaged bacteria or surrounding immune cells may release signaling molecules that trigger the death or inhibit the proliferation of nearby bystander bacteria (23). This combined approach ensures that bactericidal effects are confined to the site of infection, minimizing collateral damage to healthy tissues, and proves effective against antibiotic-resistant strains, enhancing the host immune response and overall treatment efficacy. Nonetheless, localized radiation of RIT delivery may expose adjacent immune cells to low-level radiation. Although this raises potential concerns about DNA damage, current literature suggests that immune cells can recover from controlled radiation doses to support manageable toxicity under optimized therapeutic dosing (24). Supporting this, the work of Dadachova et al., demonstrated that RIT effectively kills pathogenic fungal cells without harming nearby mammalian cells, including macrophages, further validating the safety of RIT in infectious contexts (25).

RIT offers targeted therapy, which has advantages but also potential risks and concerns. The primary concern is related to the use of radioisotopes, which may expose healthy cells to radiation and increase the risk of developing secondary cancers in patients. However, alpha particles typically require only one or two hits to destroy host cells (26), so RIT is unlikely to cause significant late-phase cytogenic effects. In addition, our team analyzed the biodistribution of ^111^Indium labeled 4497-IgG1 in mice and showed high uptake at the infected site within 120 h (11). The 4497-IgG1 compound was found to have a high uptake in organs with high blood flow, such as the heart, liver, lungs and kidneys, likely due to hepatic clearance rather than non-specific absorption, and these antibodies were rapidly cleared from non-target areas. Over time, it demonstrated a strong preference for infected implantation sites and displayed a favorable pattern of distribution throughout the body. However, extended exposure to the high activity of long-lived radionuclides used in radioimmunotherapy may present a risk of toxicity to healthy tissues due to slow clearance. Radiation therapy can potentially cause myelosuppression, which is a condition that reduces bone marrow activity, resulting in a decrease in blood cell counts. This condition can increase the risk of bleeding and infections (27). However, phase 1/2 trials have shown that isotopes like ^225^Ac can be safely managed with optimized dosing (28, 29). A study by Kozak et al., found that even at high doses of up to 1185 MBq/kg of ^177^Lu labeled mAb BR96, immunocompetent rats did not experience severe adverse effects (30). Although there were dose-dependent decreases in leukocytes and platelets, both blood counts recovered within 2 weeks. This suggests that it may be possible to increase the therapeutic dose without significantly affecting healthy tissue toxicity. It is crucial to ensure the safety and efficacy of radiopharmaceuticals in animal models before proceeding to clinical trials in the future.

The translational potential of RIT in the treatment of PJIs lies in its role as a complementary therapy, especially in cases where antibiotics alone are ineffective due to resistance. Given the severe outcomes of PJIs, which carry a mortality rate similar to certain cancers (1, 2), the benefits of RIT may outweigh the risks of radiation for patients with limited treatment options. Optimizing dose and targeting to minimize exposure to healthy tissues is an ongoing focus to make RIT a safe and effective adjunct in the clinical management of complex, antibiotic-resistant infections.

In conclusion, our study illustrates the potent antibacterial activity of [^225^Ac]4497-IgG1 and [^177^Lu]4497-IgG1 against both planktonic and biofilm-associated *S. aureus in vitro*. These findings suggest that radioimmunotherapy holds promise as a novel and effective therapeutic approach for tackling stubborn biofilm-associated infections. Further, *in vivo* studies are necessary to confirm these results and to evaluate the safety and efficacy of this approach in clinical settings.

## Data Availability

The raw data supporting the conclusions of this article will be made available by the authors, without undue reservation.
