# Supplementary material for: Radioimmunotherapy combating biofilm-associated infection in vitro
Source: Front Med (Lausanne). 2024 Nov 29;11:1478636. doi: 10.3389/fmed.2024.1478636 (PMC11637858; doi:10.3389/fmed.2024.1478636)
Supplement: Supplementary file 1 [file Data_Sheet_1.docx]

Supplementary Material

## Supplementary Figures
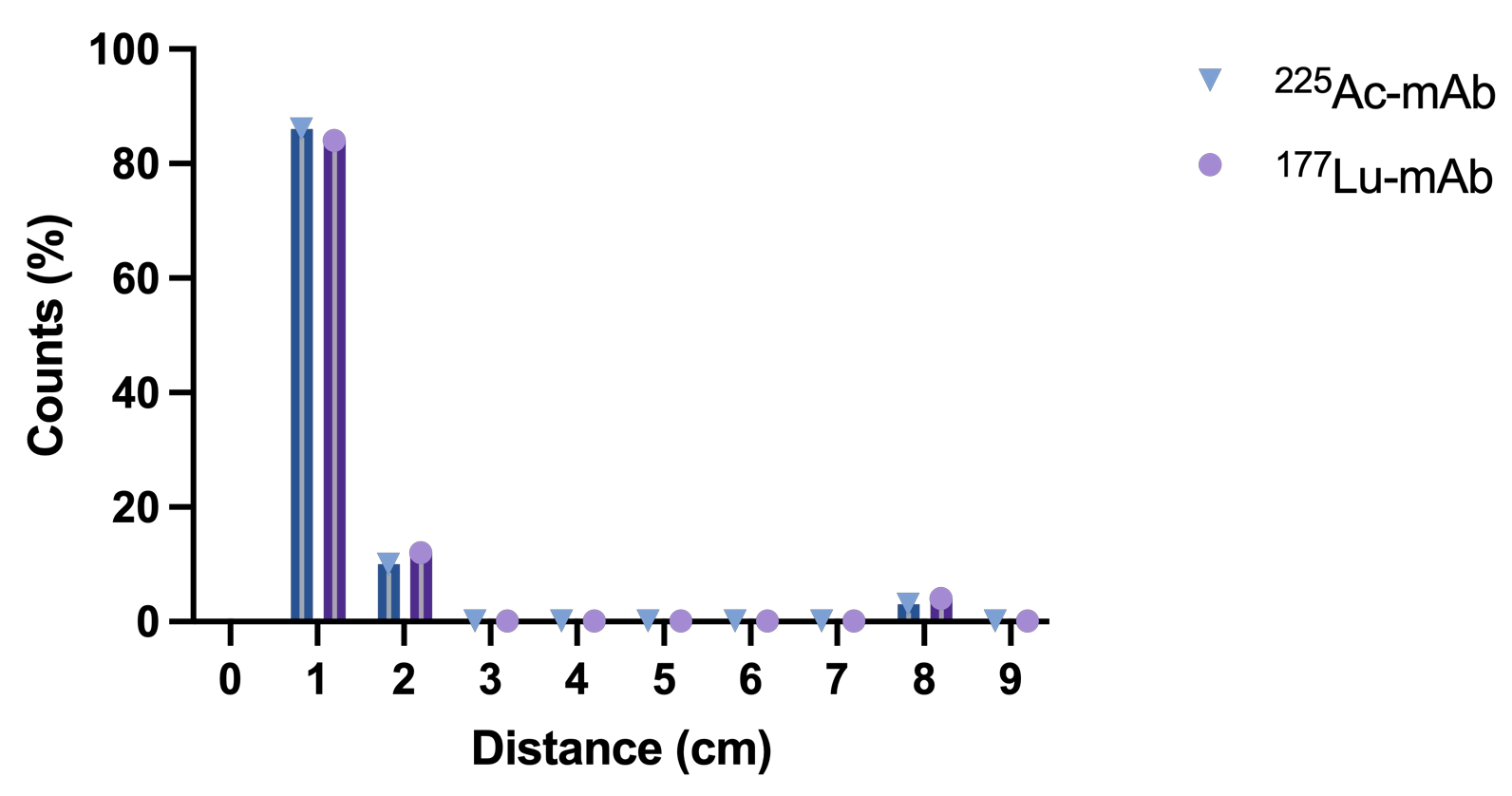


**Supplementary Figure 1.** Gamma counts recorded from different parts of the chromatographic paper relative to the distance from the bottom of the paper.
